# Response to Electrostimulation Is Impaired in Muscle Cells from Patients with Chronic Obstructive Pulmonary Disease

**DOI:** 10.3390/cells10113002

**Published:** 2021-11-03

**Authors:** Matthias Catteau, Emilie Passerieux, Léo Blervaque, Farés Gouzi, Bronia Ayoub, Maurice Hayot, Pascal Pomiès

**Affiliations:** 1 PhyMedExp, University of Montpellier—INSERM—CNRS, 34295 Montpellier, France; matthias.catteau@gmail.com (M.C.); emilie.passerieux@inserm.fr (E.P.); leo.blervaque@gmail.com (L.B.); 2 PhyMedExp, University of Montpellier—INSERM—CNRS—CHRU Montpellier, 34295 Montpellier, France; f-gouzi@chu-montpellier.fr (F.G.); b-ayoub@chu-montpellier.fr (B.A.); m-hayot@chu-montpellier.fr (M.H.)

**Keywords:** EPS, satellite cells, skeletal muscle, COPD, muscle weakness, differentiation, mitochondrial biogenesis

## Abstract

Among the comorbidities associated with chronic obstructive pulmonary disease (COPD), skeletal muscle weakness and atrophy are known to affect patient survival rate. In addition to muscle deconditioning, various systemic and intrinsic factors have been implicated in COPD muscle dysfunction but an impaired COPD muscle adaptation to contraction has never been extensively studied. We submitted cultured myotubes from nine healthy subjects and nine patients with COPD to an endurance-type protocol of electrical pulse stimulation (EPS). EPS induced a decrease in the diameter, covered surface and expression of MHC1 in COPD myotubes. Although the expression of protein degradation markers was not affected, expression of the protein synthesis marker mTOR was not induced in COPD compared to healthy myotubes after EPS. The expression of the differentiation markers p16^INK4a^ and p21 was impaired, while expression of Myf5 and MyoD tended to be affected in COPD muscle cells in response to EPS. The expression of mitochondrial biogenesis markers PGC1α and MFN2 was affected and expression of TFAM and COX1 tended to be reduced in COPD compared to healthy myotubes upon EPS. Lipid peroxidation was increased and the expression of the antioxidant enzymes SOD2 and GPx4 was affected in COPD compared to healthy myotubes in response to EPS. Thus, we provide evidence of an impaired response of COPD muscle cells to contraction, which might be involved in the muscle weakness observed in patients with COPD.

## 1. Introduction

Chronic obstructive pulmonary disease (COPD) has become one of the leading causes of death in the world and is characterized by a progressive airflow obstruction from inhaled toxic particles. Comorbidities are often associated with COPD and among them muscle weakness and atrophy are known to affect patient quality of life and survival rate [1,2]. Muscle weakness, a common characteristic of the lower limbs of patients with COPD, is associated with reduced muscle strength and endurance. Although muscle deconditioning is often cited to explain muscle dysfunction in COPD, a complex interplay of various systemic and intrinsic factors can also be advanced. Systemic inflammation [3], oxidative stress [4], impaired capillary remodeling [5] and reduced cortical excitability [6] have been shown to be implicated in COPD muscle dysfunction. However, among the combination of different factors contributing to the muscle dysfunction in these patients, an altered muscle response to contraction, indicating impaired muscle adaptation to exercise, has not been extensively studied.

In healthy subjects, muscle adaptation to exercise leads to the activation of a variety of signaling pathways. Among them, the serine/threonine kinase mTOR is a central component of the AKT/mTOR protein synthesis pathway leading to hypertrophy [7], as well as an activator of myogenesis through activation of the myogenic transcription factor MyoD [8,9]. Muscle activity also leads to increased expression of the co-activator of transcription factors PGC1α, a positive regulator of mitochondrial oxidative metabolism. Indeed, PGC1α is involved in mitochondrial biogenesis through TFAM-dependent expression of mitochondrial OXPHOS proteins and MFN2-dependent mitochondrial fusion [10], as well as in the activation of the antioxidant response [11].

Over the years, electrical pulse stimulation (EPS) has been used as an in vitro exercise model to mimic in vivo muscle adaptations to both endurance and resistance training. By combining different pulse stimulation variables, two major in vitro EPS protocols have emerged. Low-frequency stimulations over a long period correspond to in vivo endurance training, whereas high-frequency EPS over a short period corresponds to resistance training. Indeed, a chronic EPS protocol was found to increase glucose and fatty acid oxidation, mitochondrial content and MHC1 expression in human skeletal muscle cells [12] and to enhance PGC1α, mitochondrial OXPHOS proteins and MHC1 expression, and fatty acid and glucose metabolism in the mouse skeletal muscle C2C12 cell line [13]. In contrast, an acute EPS protocol in human primary myotubes activated glucose uptake [12] and the protein synthesis pathway via phosphorylation of AKT, mTOR and p70S6K [14,15]. Interestingly, the addition of a resting period after a mixed short-term/low-frequency EPS protocol induced the hypertrophy of cultured human myotubes [16].

Using an in vitro cellular model, we have recently shown that myotubes from patients with COPD have characteristics similar to those of in vivo COPD skeletal muscles. In particular, COPD myotubes exhibit an atrophy due to increased protein degradation in relation to elevated oxidative stress [17,18,19]. In this study, we used this in vitro model of the COPD muscle alteration to investigate the intrinsic response of COPD myotubes to an endurance-type EPS protocol. Pathways related to the muscle-adaptive response to exercise, such as muscle fiber growth, differentiation, mitochondrial biogenesis and antioxidant defense, were compared between COPD and healthy myotubes in response to contraction.

## 2. Materials and Methods

### 2.1. Study Population

Sedentary healthy subjects and stable patients with COPD were recruited in previous studies with no link to the present study [18,20,21]. The institutional ethics committees of the Montpellier University Hospital (Comité de Protection des Personnes Sud Méditerranée IV 2008-03-ESSS-V2, 2009-04-BPCO-V2, 2011-A00842-39 and 2013-A01199-36) approved the studies. The subjects, who had participated in the earlier studies, all gave written consent at that time to having their tissue samples used in complementary studies in addition to the initial studies. All research protocols were conducted in accordance with the good clinical practices detailed in the Helsinki Declaration and the European Guidelines.

The healthy subjects (n = 9) were recruited based on the following criteria: no disease and less than 150 min of moderate-to-vigorous physical activity per week. The patients with COPD (n = 9) were recruited on the basis of dyspnea, chronic cough and/or sputum production, and/or a history of exposure to risk factors for the disease, and a post-broncho-dilatator forced expiratory volume in 1 s/forced vital capacity ratio (FEV1/FVC), assessed by spirometry, lower than 70%. Exacerbations within the last month were exclusion criteria. Body composition and nutritional status were evaluated with the fat-free-mass index (FFMI) and the body mass index (BMI). A 6-min walking test was performed in a 30-m corridor and the 6-min walking distance (6MWD) was compared with reference values [22]. The quadriceps muscle voluntary contraction (qMVC) was assessed with the usual methods of our group [23].

### 2.2. Muscle Biopsy and Myoblast Purification

Biopsies were performed using the needle methodology in the vastus lateralis of the quadriceps of healthy subjects and patients with COPD described previously [24]. Briefly, after local anesthesia, the quadriceps muscle was incised with a scalpel and a piece of fresh biopsy was obtained with a biopsy needle. To preserve cell integrity, the biopsy samples were progressively frozen to −80 °C for 24 h in a Mr. Frosty freezing container (Nalgene Fisher Scientific, Pittsburgh, PA, USA) and then placed in liquid nitrogen. Myoblasts were purified from the frozen muscle biopsies using an anti-CD56 antibody (BD Biosciences, Franklin Lakes, NJ, USA) and an immunomagnetic sorting system (Miltenyi Biotec, Bergisch Gladbach, Germany) as described in detail previously [18].

### 2.3. Cell Culture and Electrical Pulse Stimulation

Myoblasts were grown in proliferation medium (DMEM/20% FBS/0.5% Ultroser) in collagen-coated Petri dishes. Myoblast cultures were used in all experiments at a passage below P4. When myoblasts reached 80% confluence, myogenic differentiation was induced by switching to differentiation medium (DMEM/2% FBS). Myotubes were grown for 4 days in differentiation medium. Myotubes were then subjected, or not, to EPS for the last 24 h. More precisely, continuous EPS was performed for 24 h with a C-Pace EP cell culture stimulator (IonOptix, Dublin, Ireland) on cells plated on collagen-coated 6-well plates with a protocol consisting of 4 ms pulses at 11.5 volts with a frequency of 1 Hz.

### 2.4. Fluorescence Microscopy

Immunofluorescence microscopy was performed as described previously [25]. Briefly, myotubes were fixed in PBS/3.7% paraformaldehyde, permeabilized with PBS/0.1% Triton X-100 and then incubated with a mouse monoclonal anti-troponin T antibody 1/200 (Sigma-Aldrich St. Louis, MO, USA) for 1 h at 37 °C, followed by an anti-mouse antibody coupled with Alexa Fluor 546 (Sigma-Aldrich) and Hoechst 33258 (Sigma-Aldrich) for 1 h at 37 °C. Images were captured at a 5× magnification on a Zeiss AxioImager M1 Microscope (CarlZeiss, Oberkochen, Germany) coupled with an AxioCam MRm CCD camera (CarlZeiss) driven by AxioVision 4 software (CarlZeiss). The myotube diameter, covered surface and fusion index were assessed from 9 anonymized images using ImageJ software (National Institutes of Health, Bethesda, MD, USA). For diameter analysis, 180 myotubes per condition were analyzed by taking 3 measures of the diameter along each myotube. One value, corresponding to the average diameter of the myotubes, was taken into account for each condition. The myotube-covered surface was determined by quantifying the percentage of the surface covered by myotubes per field per condition. To determine the fusion index, the number of nuclei contained in the myotubes was counted over the total number of nuclei per each image, with at least 5000 nuclei counted per condition.

### 2.5. SDS-PAGE and Immunoblotting

**Figure 1:**
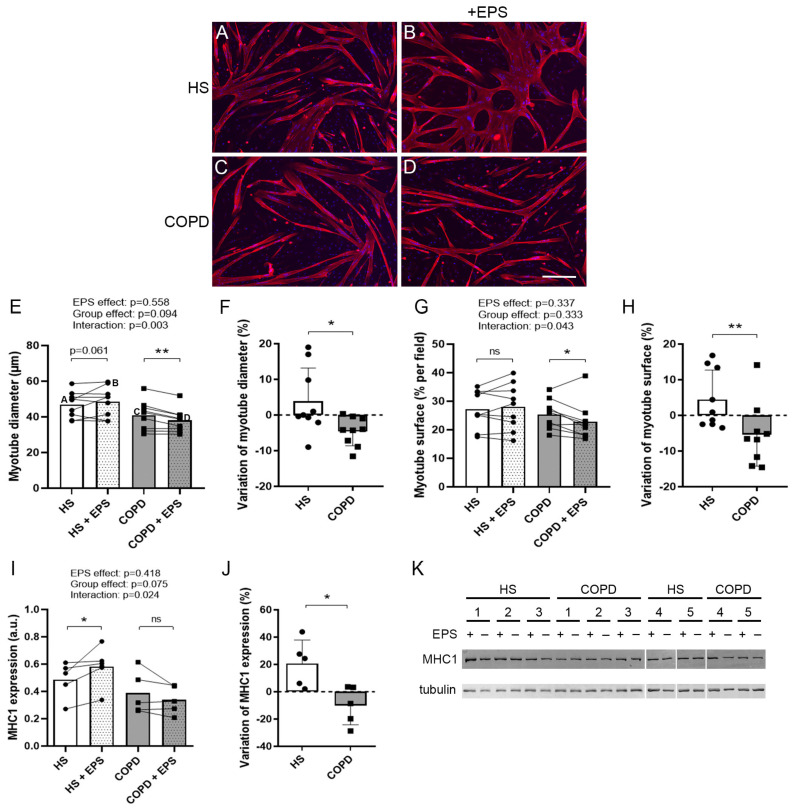
**Morphological characteristics of healthy and COPD myotubes after EPS.** (**A**–**D**) Representative images of myotubes from healthy subjects (HS) and patients with COPD (COPD) subjected (+EPS) or not to EPS. Staining: troponin-T (red) and Hoechst (blue). Bar = 200 µm. (**E**) Myotube diameter, (**G**) myotube surface and (**I**) expression levels of MHC1 were quantified from cell cultures from healthy subjects (HS) and patients with COPD (COPD) subjected (+EPS) or not to EPS. Lines link muscle cell cultures from the same subject. The means are indicated and the letters correspond to the myotube cultures presented in Figure 1A–D. *p*-values of EPS effect, Group effect and Interaction (EPS x Group) are indicated. Repeated measure two-way ANOVA followed by a Fisher’s LSD multiple comparison test was used. a.u. = arbitrary unit. The variation in the (**F**) myotube diameter, (**H**) myotube surface and (**J**) expression levels of MHC1 of healthy (HS) and COPD myotube cultures between +EPS and no EPS is presented. Data are expressed in mean ± SD. The percentages of variation were compared using a Mann-Whitney test. (**K**) Representative Western blots, quantified and analyzed in (**I**), showing the expression levels of MHC1 and tubulin in cultured myotubes from 5 healthy subjects (HS) or 5 patients with COPD (COPD) subjected (+) or not (-) to EPS. (*) and (**) indicate statistical significance at *p* < 0.05 and *p* < 0.01, respectively, and (ns) indicates statistically non-significant. n = 9 (**E**–**H**) or 5 (**I**,**J**) (HS and COPD).

Proteins were extracted from the myotubes using the NucleoSpin RNA/protein purification kit (Macherey-Nagel, Düren, Germany) according to the manufacturer’s protocol. Proteins were separated by SDS-PAGE and transferred to Immobilon-P PVDF membranes (Millipore, Bedford, MA, USA). A protein extract was loaded on each gel as a calibrator but this calibrator lane was deleted for better visualization of the presented data in Figure 1 and Figures 3 and 6. Proteins of interest were revealed by specific antibodies: anti-α-tubulin 1/20,000 (Sigma-Aldrich); anti-MHC1 1/2000 (Sigma-Aldrich); anti-phospho-AKT 1/2000 (Cell Signaling Technology, Danvers, MA, USA); anti-AKT 1/2000 (Cell Signaling Technology), followed by a secondary antibody 1/30,000 (Eurobio Scientific, Les Ulis, France). Lipid peroxidation was detected by anti-4-hydroxynonenal antibody 1/400 (Millipore, Bedford, MA, USA). The membranes were analyzed on an Odyssey imaging system (LI-COR Biotechnology, Lincoln, NE) and the band densities were quantified using ImageJ software (National Institutes of Health).

### 2.6. Quantitative Polymerase Chain Reaction

Total RNA was extracted from the myotubes using the NucleoSpin RNA/protein purification kit (Macherey-Nagel, Düren, Germany) according to the manufacturer’s protocol. The RNA was reverse transcribed using the Verso cDNA Synthesis Kit (Fisher Scientific, Illkirch, France). Quantitative PCR was performed in triplicate wells with gene-specific primers using the LightCycler 480 system (Roche, Basel, Switzerland) and SYBR Green 1 Master Mix (Roche) as follows: 40 cycles of amplification with 10 s at 95 °C, 20 s at 60 °C and 20 s at 72 °C. The list of the primers used in this manuscript is presented in Table S1. Ct values of the target gene were normalized to the Ct values of the housekeeping gene RPLP0. The expression level of each transcript was determined using the 2^−ΔΔCt^ method.

### 2.7. Statistical Analysis

Statistical analyses were performed with GraphPad Prism 8 software. Repeated measure two-way ANOVA followed by a Fisher’s LSD multiple comparison test was used to compare values between control and EPS conditions for healthy and COPD myotubes for the various studied variables. The percentages of variation of the various parameters for healthy and COPD myotubes were compared using a Mann-Whitney test. Spearman’s coefficient was used to assess correlations between variables. Significance has been set at *p* < 0.05.

## 3. Results

### 3.1. Characteristics of the Study Groups

The main clinical and functional characteristics of the two study groups, healthy subjects (n = 9) and patients with COPD (n = 9), are presented in Table 1. FEV1, 6MWD and qMVC values indicated severely impaired lung function associated with a moderate exercise limitation and muscle dysfunction in the patients.

### 3.2. Effects of EPS on Myotube Morphology

Myotube cultures from the healthy subjects and patients with COPD were subjected to 24 h EPS. Representative images of the cultured myotubes are shown in Figure 1A–D. EPS tended to increase the healthy myotube diameters (*p* = 0.061) and significantly reduced the COPD myotube diameters (*p* = 0.005), with a significant between-group difference (interaction: *p* = 0.003; Figure 1E). In addition, the variation in myotube diameters after EPS was significantly different (*p* = 0.026; Figure 1F) between healthy myotubes (+3.8%) and COPD myotubes (−4.6%). Similarly, the surface covered by myotubes was unchanged for healthy myotubes and reduced for COPD myotubes (*p* = 0.035) after EPS (interaction: *p* = 0.043; Figure 1G). EPS-induced variations in the surface covered by myotubes were significantly different (*p* = 0.009; Figure 1H) for healthy (+4.5%) and COPD myotubes (−5.3%). Moreover, EPS induced an increase in the expression of the structural protein MHC1 in healthy myotubes (*p* = 0.033) but not in COPD myotubes, with a significant between-group difference (interaction: *p* = 0.024; Figure 1I). The variations in MHC1 expression were also significantly different (*p* = 0.031; Figure 1J) between healthy (+20.8%) and COPD myotubes (−10.1%).

### 3.3. Effects of EPS on Protein Degradation and Synthesis Pathways

**Figure 2:**
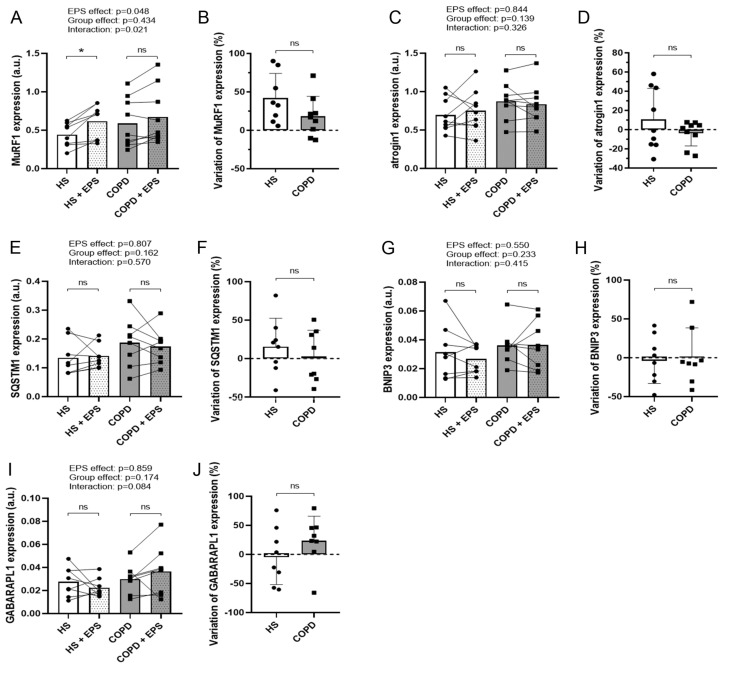
**Expression levels of protein degradation markers in healthy and COPD myotubes after EPS.** Analysis of the expression levels of (**A**) MuRF1, (**C**) atrogin1, (**E**) SQSTM1, (**G**) BNIP3 and (**I**) GABARAPL1 in myotube cultures from healthy subjects (HS) and patients with COPD (COPD) subjected (+EPS) or not to EPS. Lines link muscle cell cultures from the same subject and the means are indicated. *p*-values of EPS effect, Group effect and Interaction (EPS × Group) are indicated. Repeated measure two-way ANOVA followed by a Fisher’s LSD multiple comparison test was used. a.u. = arbitrary unit. The variation in the expression levels of (**B**) MuRF1, (**D**) atrogin1, (**F**) SQSTM1, (**H**) BNIP3 and (**J**) GABARAPL1 in myotube cultures from healthy subjects (HS) and patients with COPD (COPD) between +EPS and no EPS is presented. Data are expressed in mean ± SD. The percentages of variation were compared using a Mann-Whitney test. (*) indicates statistical significance at *p* < 0.05 and (ns) indicates statistically non-significant. n = 8 or 9 (HS and COPD).

**Figure 3:**
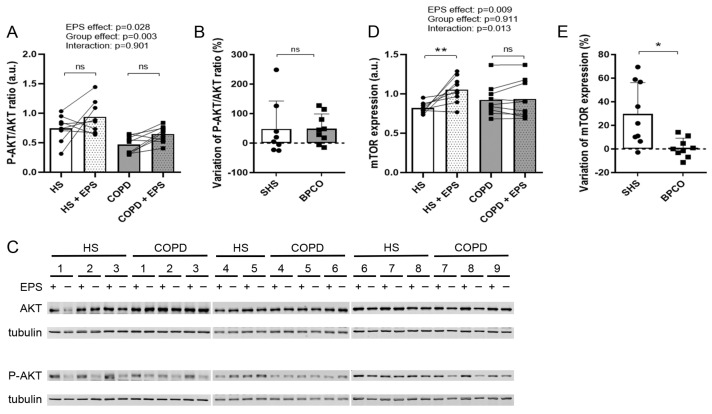
**Expression levels of protein synthesis markers in healthy and COPD myotubes after EPS.** Analysis of (**A**) the phospho-AKT/AKT ratio and (**D**) mTOR expression levels in myotube cultures from healthy subjects (HS) and patients with COPD (COPD) subjected (+EPS) or not to EPS. Lines link muscle cell cultures from the same subject and the means are indicated. *p*-values of EPS effect, Group effect and Interaction (EPS × Group) are indicated. Repeated measure two-way ANOVA followed by a Fisher’s LSD multiple comparison test was used. a.u. = arbitrary unit. The variation in (**B**) the phospho-AKT/AKT ratio and (**E**) mTOR expression levels in myotube cultures from healthy subjects (HS) and patients with COPD (COPD) between +EPS and no EPS is presented. Data are expressed in mean ± SD. The percentages of variation were compared using a Mann-Whitney test. Data are expressed in mean ± SD. The percentages of variation were compared using a Mann-Whitney test. (**C**) Representative Western blots, quantified and analyzed in (**A**), showing the expression levels of AKT, phospho-AKT and tubulin in cultured myotubes from 8 healthy subjects (HS) or 9 patients with COPD (COPD) subjected (+) or not (-) to EPS. (*) and (**) indicate statistical significance at *p* < 0.05 and *p* < 0.01, respectively, and (ns) indicates statistically non-significant. n = 8 or 9 (HS and COPD).

As EPS had opposite effects on the morphology of the healthy and COPD myotubes, we studied the expression of various markers of protein breakdown and protein synthesis pathways. EPS induced an increase in MuRF1 expression in both groups (EPS effect: *p* = 0.048; Figure 2A), whereas variations in the expression of MuRF1 and atrogin1 were similar in the two groups after EPS (Figure 2B,D). No difference in the variations in expression of autophagy markers SQSTM1, BNIP3 and GABARAPL1 was observed after EPS between healthy and COPD myotubes (Figure 2F,H,J). EPS induced an increase in the P-AKT/AKT ratio in both healthy and COPD myotubes (EPS effect: *p* = 0.028; Figure 3A), and the variations in this ratio were similar in the two groups (Figure 3B). Interestingly, EPS significantly increased mTOR expression in healthy myotubes (*p* = 0.001) but not in COPD myotubes (Interaction: *p* = 0.013; Figure 3D), and the variations in mTOR expression were significantly different (*p* = 0.010; Figure 3E) between the healthy myotubes (+29.7%) and COPD myotubes (+0.9%).

### 3.4. Effects of EPS on Differentiation Marker Expression

**Figure 4:**
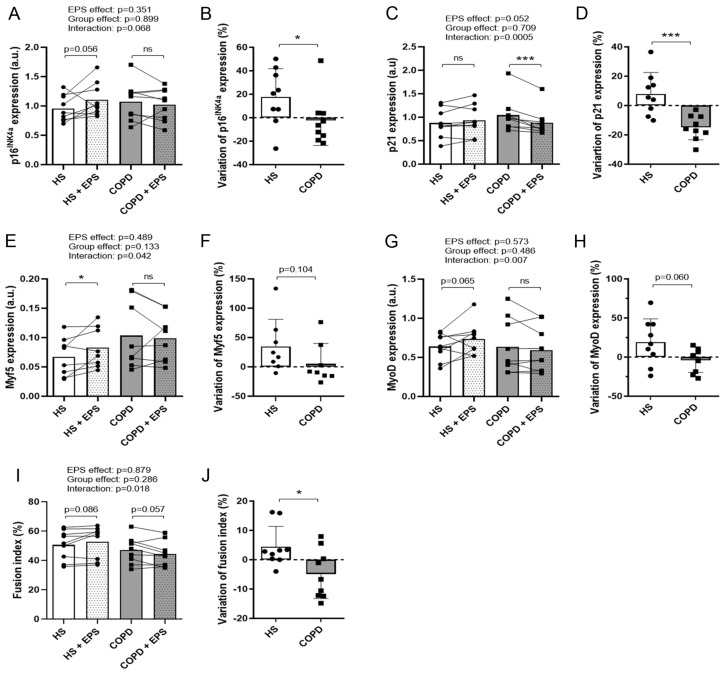
**Expression levels of differentiation markers in healthy and COPD myotubes after EPS.** Analysis of the expression levels of (**A**) p16^INK4a^, (**C**) p21, (**E**) Myf5 and (**G**) MyoD, and of the myotube fusion index (**I**) in myotube cultures from healthy subjects (HS) and patients with COPD (COPD) subjected (+EPS) or not to EPS. Lines link muscle cell cultures from the same subject and the means are indicated. *p*-values of EPS effect, Group effect and Interaction (EPS × Group) are indicated. Repeated measure two-way ANOVA followed by a Fisher’s LSD multiple comparison test was used. a.u. = arbitrary unit. The variation in the expression levels of (**B**) p16^INK4a^, (**D**) p21, (**F**) Myf5 and (**H**) MyoD, and of the myotube fusion index (**J**) in myotube cultures from healthy subjects (HS) and patients with COPD (COPD) between +EPS and no EPS is presented. Data are expressed in mean ± SD. The percentages of variation were compared using a Mann-Whitney test. (*) and (***) indicate statistical significance at *p* < 0.05 and *p* < 0.001, respectively, and (ns) indicates statistically non-significant. n = 8 or 9 (HS and COPD).

Expression of p16^INK4a^ and p21, two positive regulators of muscle early differentiation [26,27], was assessed in healthy and COPD myotubes after EPS. Variations in p16^INK4a^ expression were significantly different between the two groups (*p* = 0.050; Figure 4B), with enhanced expression in healthy myotubes (+17.7%) and decreased expression in COPD myotubes (−2.4%). A significant difference in the variations in p21 expression was observed in the two groups after EPS (*p* = 0.0008; Figure 4D), with increased expression in healthy myotubes (+8.0%) and reduced expression in COPD myotubes (−14.9%). Furthermore, expression of the differentiation marker Myf5 increased significantly in healthy myotubes (*p* = 0.028) but not in COPD myotubes after EPS (interaction: *p* = 0.042; Figure 4E). In a similar way, MyoD expression tended to increase in healthy myotubes after EPS (*p* = 0.065) but not in COPD myotubes, with a significant difference between groups (interaction: *p* = 0.007; Figure 4G). As a result of this enhanced expression of differentiation markers observed in healthy but not in COPD myotubes, the fusion index was significantly improved (interaction: *p* = 0.018, Figure 4I; *p* = 0.042, Figure 4J) in the healthy myotubes (+4.4%) compared to COPD myotubes (−4.9%) after EPS.

### 3.5. Effects of EPS on Mitochondrial Biogenesis

**Figure 5:**
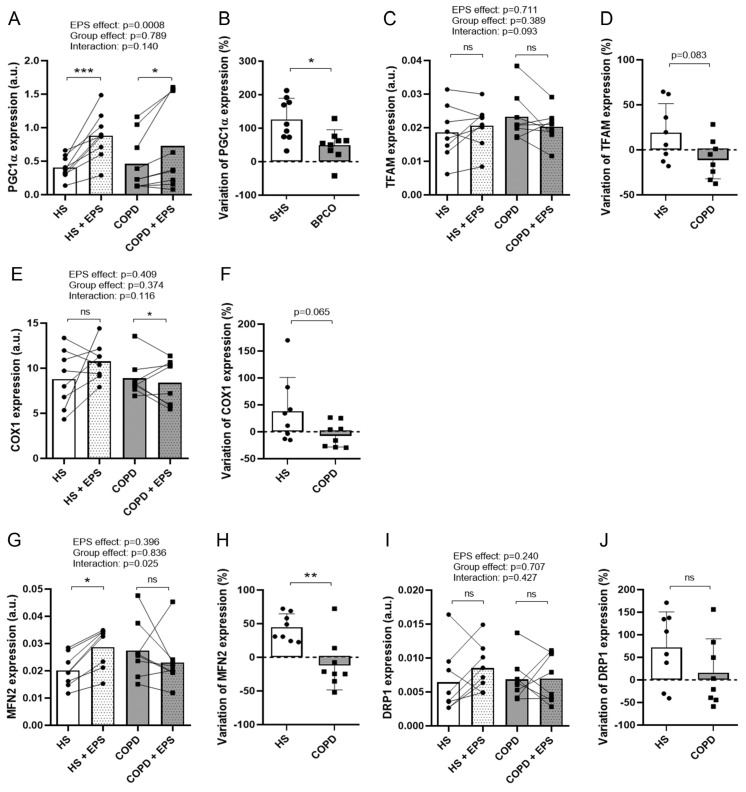
**Expression levels of mitochondrial markers in healthy and COPD myotubes after EPS.** Analysis of the expression levels of (**A**) PGC1α, (**C**) TFAM, (**E**) COX1, (**G**) MFN2 and (**I**) DRP1 in myotube cultures from healthy subjects (HS) and patients with COPD (COPD) subjected (+EPS) or not to EPS. Lines link muscle cell cultures from the same subject and the means are indicated. *p*-values of EPS effect, Group effect and Interaction (EPS × Group) are indicated. Repeated measure two-way ANOVA followed by a Fisher’s LSD multiple comparison test was used. a.u. = arbitrary unit. The variation in the expression levels of (**B**) PGC1α, (**D**) TFAM, (**F**) COX1, (**H**) MFN2 and (**J**) DRP1 in myotube cultures from healthy subjects (HS) and patients with COPD (COPD) between +EPS and no EPS is presented. Data are expressed in mean ± SD. The percentages of variation were compared using a Mann-Whitney test. (*), (**) and (***) indicate statistical significance at *p* < 0.05, *p* < 0.01 and *p* < 0.001, respectively, and (ns) indicates statistically non-significant. n = 9 (**A**,**B**) or 8 (**C**–**J**) (HS and COPD).

EPS significantly enhanced PGC1α expression in both groups (EPS effect: *p* = 0.0008; Figure 5A), but variations in PGC1α expression were significantly higher (*p* = 0.014; Figure 5B) in healthy myotubes (+126.0%) compared to COPD myotubes (+49.7%). Variations in TFAM expression tended to be different between the two groups (*p* = 0.083; Figure 5D) with enhanced expression in healthy myotubes (+19.0%) and reduced expression in COPD myotubes (−9.8%). Expression of COX1 was unchanged for healthy myotubes and reduced for COPD myotubes (*p* = 0.044; Figure 5E) after EPS, while variations in COX1 expression tended to be different (*p* = 0.065; Figure 5F) between the healthy myotubes (+38.5%) and COPD myotubes (−5.0%). No difference in the variations of expression of the other respiratory chain markers ND2, CYTB and ATP6 was observed after EPS between healthy and COPD myotubes (Figure S1). While EPS had no effect on the expression of the mitochondrial fission marker DRP1 (Figure 5I,J), expression of the fusion marker MFN2 increased significantly in healthy myotubes (*p* = 0.031; Figure 5G) but not in COPD myotubes after EPS (interaction: *p* = 0.025; Figure 5G). Moreover, the variations in MFN2 expression were significantly different (*p* = 0.007; Figure 5H) between the healthy myotubes (+44.6%) and COPD myotubes (−10.1%).

### 3.6. Effects of EPS on Oxidative Stress and Antioxidant Enzyme Expression

**Figure 6:**
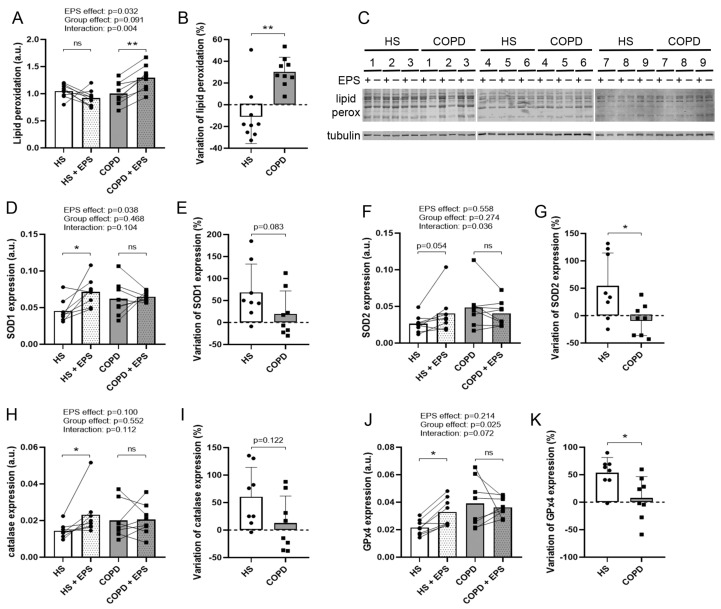
**Lipid peroxidation levels and expression of antioxidant enzymes in healthy and COPD myotubes after EPS.** (**A**) Analysis of the lipid peroxidation levels in myotubes from healthy subjects (HS) and patients with COPD (COPD) subjected (+EPS) or not to EPS. Lines link muscle cell cultures from the same subject and the means are indicated. *p*-values of EPS effect, Group effect and Interaction (EPS × Group) are indicated. Repeated measure two-way ANOVA followed by a Fisher’s LSD multiple comparison test was used. a.u. = arbitrary unit. The variation in lipid peroxidation in myotube cultures from healthy subjects (HS) and patients with COPD (COPD) between +EPS and no EPS is presented (**B**). (**C**) Representative Western blots, quantified and analyzed in (**A**), showing the lipid peroxidation levels and tubulin expression levels in cultured myotubes from 9 healthy subjects (HS) or 9 patients with COPD (COPD) subjected (+) or not (-) to EPS. Analysis of the expression levels of (**D**) SOD1, (**F**) SOD2, (**H**) catalase and (**J**) GPx4 in myotube cultures from healthy subjects (HS) and patients with COPD (COPD), subjected (+EPS) or not to EPS. The variation in the expression levels of (**E**) SOD1, (**G**) SOD2, (**I**) catalase and (**K**) GPx4 in myotube cultures from healthy subjects (HS) and patients with COPD (COPD) between +EPS and no EPS is presented. Data are expressed in mean ± SD. The percentages of variation were compared using a Mann-Whitney test. (*) and (**) indicate statistical significance at *p* < 0.05 and *p* < 0.01, respectively, and (ns) indicates statistically non-significant. n = 9 (**A**–**C**) or n = 8 (**D**–**K**) (HS and COPD).

Lipid peroxidation was unchanged for healthy myotubes and increased for COPD myotubes (*p* = 0.005) after EPS (interaction: *p* = 0.004; Figure 6A). In addition, the variation in lipid peroxidation after EPS was significantly different (*p* = 0.002; Figure 6B) between healthy myotubes (−10.2%) and COPD myotubes (+30.3%). SOD1 and catalase expressions were significantly enhanced in healthy myotubes (*p* = 0.021, *p* = 0.030, respectively) but not in COPD myotubes after EPS (Figure 6D,H), and the variations in their expression tended to be higher (*p* = 0.083, *p* = 0.122, respectively; Figure 6E,I) in healthy myotubes (+68.6%, +60.4%, respectively) compared to COPD myotubes (+19.7%, +12.5%, respectively). SOD2 expression tended to increase after EPS in healthy myotubes (*p* = 0.054) but not in COPD myotubes, with a significant between-group difference (*p* = 0.036; Figure 6F). The variations in the expression of SOD2 were significantly different between the two groups (*p* = 0.036; Figure 6G), with enhanced expression in healthy myotubes (+54.4%) and decreased expression in COPD myotubes (−7.6%). The expression of GPx4 was significantly increased in healthy myotubes after EPS (*p* = 0.046; Figure 6J), and variations in GPx4 expression were significantly different (*p* = 0.020; Figure 6K) between healthy myotubes (+53.5%) and COPD myotubes (+7.8%).

## 4. Discussion

In this study, we investigated for the first time the molecular response of myotubes from patients with COPD to EPS. In contrast to myotubes from healthy subjects, COPD myotubes showed atrophy, decreased protein synthesis, altered differentiation, impaired mitochondrial biogenesis and reduced activation of antioxidant defense. Together, our data from the COPD myotubes indicate a lack of muscle adaptation to EPS.

Different EPS protocols, combining various frequencies of stimulation and durations, have been used on cultured muscle cells. Two major types of EPS protocol, short-term/high-frequency (≤8 h, ≥30 Hz) and long-term/low-frequency (≥24 h, ≤5 Hz), have emerged [28]. The EPS protocol we have used in this study is a long-term/low-frequency protocol (24 h, 1 Hz) that has been used by other teams on both the mouse myogenic C2C12 cell line and human primary myotubes from the musculus obliquus internus abdominis of healthy subjects [12,13]. In our study, EPS on myotubes from the vastus lateralis of healthy subjects induced or tended to induce the expression of the differentiation markers p16^INK4a^, Myf5 and MyoD, the protein synthesis marker mTOR, the structural protein MHC1, the positive regulator of mitochondrial biogenesis PGC1α, the mitochondrial fusion marker MFN2 and the antioxidant enzymes SOD1, SOD2, catalase and GPx4. The stimulated expression of the slow fiber marker MHC1 and the mitochondrial biogenesis activator PGC1α is a classical feature of long-term/low-frequency EPS corresponding to the signaling pathways activated during in vivo endurance training [12,13,29]. Whereas Nikolić et al. reported an unchanged phospho-AKT/AKT ratio after a 24 h/low-frequency EPS [12], activation of the protein synthesis pathway, especially the expression of phospho-AKT, -mTOR and -p70S6K, has been described for a 6 h/low-frequency protocol on primary skeletal muscle cells [14]. Furthermore, Son et al. determined that an intermediate 1 h/10 Hz EPS protocol on C2C12 myotubes, mimicking phenotypes displayed during in vivo endurance exercise in mice, induced the expression of the myogenic transcription factors MyoD and myogenin and the antioxidant enzymes SOD3 and GPx4 [30]. Together, these results indicate that the stimulation of differentiation, mitochondrial biogenesis and antioxidant defense we observed in primary healthy myotubes upon EPS is in agreement with what has been described by other teams in cultured skeletal muscle cells and validated as a model for in vivo endurance exercise [13,28,30].

The EPS in our study tended to increase the diameter of healthy myotubes and significantly enhanced the expression of the sarcomeric protein MHC1. It has previously been shown that EPS induces the assembly of sarcomeric structures and contractile activity in association with EPS-induced Ca^2+^ transients in cultured skeletal muscle cells [29,31]. However, to our knowledge, only one study has shown the hypertrophy of skeletal muscle cells upon EPS [16]. This hypertrophy was accompanied by increased phosphorylation of two key regulators of skeletal muscle hypertrophy, mTOR and 4E-BP1. Interestingly, the enlargement of cultured myotubes was dependent on the addition of an 8-h resting period following short-term/low-frequency EPS (8 h, 1 Hz) [16]. In our study, the tendency of EPS-induced hypertrophy in the healthy myotubes was associated with increased expression of mTOR. However, addition of an 8-h resting period after EPS had no further significant effect on the EPS-induced increase in the myotube diameter (unpublished personal results).

We used a long-term/low-frequency EPS protocol (24 h, 1 Hz) in this work to mimic key features of endurance exercise on human primary myotubes from patients with COPD. These cells represent a validated in vitro model of COPD muscle alteration, displaying characteristics of atrophy and oxidative stress similar to those described in in vivo COPD limb muscles [17,18,19]. The protocol used in this study, eliciting muscle contraction and the muscle adaptation associated with exercise, revealed an altered response of COPD myotubes to EPS. Indeed, in contrast to healthy myotubes, long-term/low-frequency EPS of COPD myotubes resulted in atrophy and impaired protein synthesis, muscle differentiation, myotube fusion, mitochondrial biogenesis and antioxidant defense.

mTOR is a well-described positive regulator of protein synthesis and therefore of cell growth and hypertrophy [8]. It is also necessary for skeletal muscle differentiation through controlling the expression of myogenic transcription factors such as Myf5, MyoD and myogenin [32]. Muscle activity induces the activation of mTOR thus leading to hypertrophy and increased muscle differentiation [7]. In our study, while EPS induced an increased expression of mTOR in healthy muscle cells, contraction has no effect on mTOR expression in COPD myotubes (Figure 3). This lack of activation is associated with inhibition of mTOR-modulated signaling pathways such as muscle growth (Figure 1), differentiation (Figure 4) and fusion (Figure 4), indicating an alteration of the COPD muscle response to stimulation.

PGC1α is a key regulator of oxidative phosphorylation, mitochondrial biogenesis, fiber-type shift from glycolytic to oxidative fibers, antioxidant enzyme expression and hence of skeletal muscle oxidative capacity [33]. Moreover, PGC1α expression is activated in vivo upon endurance exercise, which in turn mediates specific gene expression during skeletal muscle adaptation to exercise [34]. It has been shown that PGC1α expression is impaired in the muscle of patients with COPD and that it might be involved in the reduced oxidative capacities of the muscles [35]. Even though the expression of PGC1α is not altered in non-stimulated COPD myotubes in vitro, we showed that its expression response is reduced upon contraction compared to healthy muscle cells (Figure 5). In addition, variation in the expression of the mitochondrial transcription factor TFAM, a downstream effector of PGC1α, and of the complex IV subunit COX1, a target gene of TFAM, tended to be reduced in COPD myotubes compared to healthy muscle cells upon EPS (Figure 5). Expression of another key regulator of mitochondrial biogenesis, the mitochondrial fusion marker MFN2, is significantly altered in COPD myotubes after EPS (Figure 5). Interestingly, antioxidant enzyme expression, a signaling pathway known to be modulated by PGC1α, is impaired in COPD myotubes upon stimulation (Figure 6). Moreover, we observed a lower expression of MHC1 in non-stimulated COPD myotubes compared to healthy myotubes (Figure 1), which could reflect the fiber-type shift, from oxidative to glycolytic fibers, observed in the vastus lateralis of patients with COPD [36]. Interestingly, the endurance-like EPS protocol used in our study induced an increased expression of the slow fiber marker MHC1 in healthy myotubes but not in COPD myotubes (Figure 1) in accordance with the effects of a moderate-intensity endurance training on the vastus lateralis of healthy subjects and patients with COPD [37]. These results indicate a PGC1α-dependent adaptation defect of the COPD muscle in response to endurance-type contraction.

Elevated oxidative stress and its involvement in muscle weakness and atrophy have been well described in the muscle of patients with COPD in vivo and in vitro [4,19,23]. In our study, oxidative stress, depicted by lipid peroxidation, was increased in COPD myotubes but not in healthy myotubes upon EPS (Figure 6). Furthermore, EPS induced SOD1, SOD2, catalase and GPx4 expression in healthy muscle cells but not in COPD muscle cells, with the variations in SOD2 and GPx4 expression being significantly different between COPD and healthy myotubes (Figure 6). Recently, an increased mitochondrial oxidative stress and an altered mitochondrial respiration were observed in the skeletal muscles of patients with COPD with a physical activity level similar to that of healthy subjects [38]. Given that GPx4 and SOD2 play a specific role in protecting mitochondria from oxidation [39,40], our results suggest that an oxidative stress-induced mitochondrial dysfunction might be involved in the muscle weakness of patients with COPD.

In vitro EPS of cultured healthy myotubes has been shown to induce cell damage that subsequently leads to increased protein synthesis and fusion of the muscle cells, through a recovery process similar to what has been described in vivo [41,42]. The altered protein synthesis, differentiation and fusion responses we observed after EPS in COPD myotubes indicate that the recovery phase associated with EPS-induced muscle damage might be impaired in the COPD muscle cells. This alteration would explain, at least in part, the in vivo muscle dysfunction observed in patients with COPD. Interestingly, an adaptation defect to muscle contraction upon EPS has also been observed in cultured myotubes from other diseased subjects. Indeed, Feng et al. showed an increase in insulin sensitivity associated with impaired lipid oxidation and oxidative capacity after EPS in myotubes from obese subjects [43]. Upon EPS, myotubes from individuals with chronic fatigue syndrome have impaired glucose uptake and reduced IL6 secretion compared to healthy myotubes [44]. Together, these studies demonstrate that EPS on primary myotubes from diseased patients provides a reliable tool for studying the intracellular mechanisms that lead to the muscle adaptive response to exercise.

Long-term/low-frequency EPS provides an in vitro system for the study of defects in muscle adaptation to muscle contraction in several diseases [43,44]. This is the first study to apply EPS to primary myotubes from patients with COPD. Here, by comparing primary muscle cells from patients with COPD to cells from healthy subjects, we showed that EPS-induced muscle contraction leads to an impaired response of COPD myotubes with abnormal differentiation, fusion, mitochondrial biogenesis, oxidative stress and antioxidant defense. Together, these results suggest that the altered adaptation of COPD muscles to contraction might be part of the muscle weakness observed in patients with COPD.

## Figures and Tables

**Table 1:** Characteristics of the study groups.

	Healthy Subjects	Patients with COPD	*p*-Value
n	9	9	-
Gender (M/F)	7/2	6/3	-
Age (yrs)	62.3 ± 6.4	58.0 ± 6.5	0.180
BMI (kg/m^2^)	25.5 ± 3.6	20.5 ± 3.1	0.006
FFMI (kg/m^2^)	18.5 ± 2.0	16.7 ± 2.2	0.110
FEV_1_ (% pred.)	102.4 ± 14.7	36.3 ± 14.6	<0.0001
6MWD (% pred.)	93.4 ± 11.8	62.5 ± 16.3	0.0003
qMVC (kg)	36.5 ± 9.7 ^1^	18.0 ± 6.9 ^2^	0.001

Definition of abbreviations: BMI = body mass index; FFMI = fat-free mass index; FEV1 = forced expiratory volume in 1 s; 6MWD = 6-min walking distance; qMVC = quadriceps muscle voluntary contraction. % pred. = % predicted; ^1^ one missing value; ^2^ two missing values.

## Data Availability

The data that support the findings of this study are available from the corresponding author upon reasonable request.

## Supplementary Materials

The following are available online at https://www.mdpi.com/article/10.3390/cells10113002/s1. Figure S1: Expression levels of mitochondrial-encoded markers in healthy and COPD myotubes after EPS, Table S1: Primer sequences.
