# Supplementary material for: Response to Electrostimulation Is Impaired in Muscle Cells from Patients with Chronic Obstructive Pulmonary Disease
Source: Cells. 2021 Nov 3;10(11):3002. doi: 10.3390/cells10113002 (PMC8616440; doi:10.3390/cells10113002)
Supplement: Supplementary file 1 [file cells-10-03002-s001.zip › Figure S1.pdf]

Figure S1

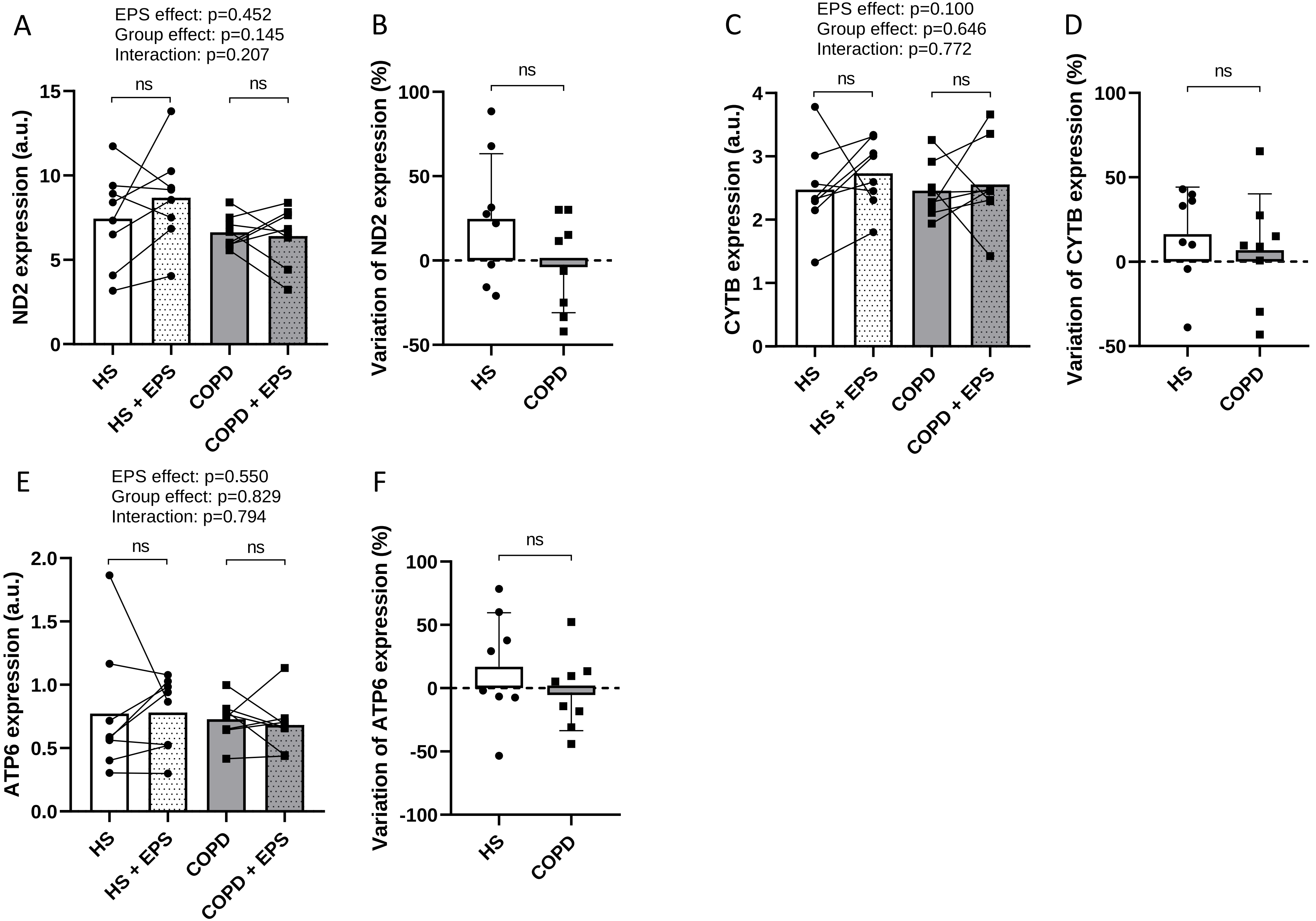
A
EPS effect: p=0.452
Group effect: p=0.145
Interaction: p=0.207
ns
ns
ND2 expression (a.u.)
15
10
5
0
HS
HS + EPS
COPD
COPD + EPS
B
ns
Variation of ND2 expression (%)
100
50
0
-50
HS
COPD
C
EPS effect: p=0.100
Group effect: p=0.646
Interaction: p=0.772
ns
ns
CYTB expression (a.u.)
4
3
2
1
0
HS
HS + EPS
COPD
COPD + EPS
D
ns
Variation of CYTB expression (%)
100
50
0
-50
HS
COPD
E
EPS effect: p=0.550
Group effect: p=0.829
Interaction: p=0.794
ns
ns
ATP6 expression (a.u.)
2.0
1.5
1.0
0.5
0.0
HS
HS + EPS
COPD
COPD + EPS
F
ns
Variation of ATP6 expression (%)
100
50
0
-50
-100
HS
COPD

Figure S1. Expression levels of mitochondrial-encoded markers in healthy and COPD myotubes after EPS. Analysis of the expression levels of (A) ND2, (C) CYTB and (E) ATP6 in myotube cultures from healthy subjects (HS) and COPD patients (COPD) subjected (+EPS) or not to EPS. Lines link muscle cell cultures from the same subject and the means are indicated. p-values of EPS effect, Group effect and Interaction (EPS x Group) are indicated. Repeated measure two-way ANOVA followed by a Fisher's LSD multiple comparison test was used. The variation in the expression levels of (B) ND2, (D) CYTB and (F) ATP6 in myotube cultures from healthy subjects (HS) and COPD patients (COPD) between +EPS and no EPS is presented. Data are expressed in mean ± SD. The percentages of variation were compared using a Mann-Whitney test. (ns) indicates statistically non-significant. n=8 (HS and COPD).
