# Supplementary material for: Response to Electrostimulation Is Impaired in Muscle Cells from Patients with Chronic Obstructive Pulmonary Disease
Source: Cells. 2021 Nov 3;10(11):3002. doi: 10.3390/cells10113002 (PMC8616440; doi:10.3390/cells10113002)
Supplement: Supplementary file 1 [file cells-10-03002-s001.zip › Table S1 NEW EPS Primers.pdf]

Table S1. Primer sequences.

Gene	Forward primer	Reverse primer
MuRF1	AAACAGGAGTGCTCCAGTCGG	CGCCACCAGCATGGAGATACA
atrogin1	CGACCTCAGCAGTTACTGCAA	TTTGCTATCAGCTCCAACAG
SQSTM1	GCACCTGTCTGAGGGCTTCT	GCTCCAGTTTCCTGGTGGAC
BNIP3	CTGAAACAGATACCCATAGCATT	CCGACTTGACCAATCCCA
GABARAPL1	GGTCCCCGTGATTGTAGAGA	GGAGGGATGGTGTTGTTGAC
mTOR	GGGGACACTTTTACCGCTGA	AATCAGACAGGCACGAAGGG
p16^{INK4a}	GAAGGTCCCTCAGACATCCCC	CCCTGTAGGACCTTCGGTGAC
p21	ACTCTCAGGGTCGAAAACGG	CTTCCTGTGGGCGGATTAGG
Myf5	TGATGGCATGCCCGAATGTA	CAGGTTGCTCTGAGGAGGTG
MyoD	ACAACGGACGACTTCTATGAC	TGCTCTTCGGGTTTCAGGA
PGC1α	ACGCACCGAAATTCTCCCTT	TCTGTCCGTGTTGTGTCAGG
TFAM	GGCACAGGAAACCAGTTAGG	CAGAACACCGTGGCTTCTAC
SOD1	GCCAAAGGATGAAGAGAGG	ACCACAAGCCAAACGAC
SOD2	AGCACGCTTACTACCTTC	CTTTCAGTTACATTCTCCCAG
catalase	CTTTGCTGAGGTTGAACAGA	CACCCTGATTGTCCTGC
GPx4	CTTCACCAAGTTCCTCATCG	GTCCTTCTCTATCACCAGGG
MFN2	CTGTGCGGAGTCAGATAGA	CATTGTAGCCCATCAAGG
DRP1	AGAACCAACCACAGGCAA	AACAGGAACTGGCACAT
